# News and Notes

**Published:** 1906-08

**Authors:** 


### NEWS AND NOTES.

---

Dr. and Mrs. W. C. Warren are in Asheville.

---

Dr. W. S. Elkin is convalescing from an attack of typhoid fever.

---

King Edward has opened a magnificent edifice built among the Sussex pine woods for the treatment of consumption.

---

Among the social diversions of the Fiftieth International Medical Congress, held at Lisbon, was a bull fight at Villa Franca.

---

Our "red back" exchange, the *Texas Medical Journal,* edited by Dr. F. E. Daniel, celebrated its twenty-first anniversary July 10th.

---

Dr. Bransford Lewis, of St. Louis, was elected president of the American Urological Association at the Boston meeting, held on June 5th.

---

Dr. W. E. Wilmerding, of Atlanta, was elected vice-president of the American Medical Examiners Association at their recent meeting in Boston.

---

There are seventeen physicians in the Russian douma besides several members who have studied medicine, but have not been officially registered.

---

The first Japanese woman who studied medicine in Germany is Miss Lada Urata, who has been made professor, *honoris causa,* by her own government.

WARRANTS were issued June 12th against twelve physicians of Birmingham, who were charged with failure to make obstetrical reports for the month of May.

---

AMONG the appropriations passed by the New York board of aldermen, on July 10th, was one of $628,000 for a training school for nurses at Bellevue Hospital.

---

ASCARELLI of Rome recently reported to the local medical society the case of a woman of thirty-eight with peuperal osteomalacia cured by Roentgen treatment.

---

THE ninety-third annual graduating exercises of Yale University medical department, New Haven, were held June 27, when a class of twenty-five was graduated.

---

STEPS have been taken to prosecute Dr. P. Mouigut, of LaPlace, La., for failing to report a case of yellow fever to the health officers until after the patient's recovery.

---

"MEDICINE," May, 1906, says: "If every medical college in the United States were to be closed for five years, it would be of advantage to the profession and to the community."

---

DR. SIDNEY O. HEISKELL, the quarantine physician for the port of Baltimore, died suddenly on Friday evening, June 22, on the lawn in front of his home at the quarantine station.

---

INVITATIONS have been issued by the president and fellows of Harvard College and the Faculty of Medicine to the dedication of the new buildings of the Harvard Medical School on Tuesday afternoon and Wednesday morning, September 25th and 26th, 1906.

DR. HENRY R. SLACK, of the LaGrange Sanatorium, has recently returned from Boston, where he took a postgraduate course at the Harvard Medical School. Mrs. Slack accompanied him.

---

THE American Medical Association, at the recent meeting in Boston, appropriated $5,000 for the physicians of San Francisco and surroundings, who were made destitute by the earthquake and fire.

---

THE medical faculty of the Paris University have planned an international technological encyclopedia, to be issued in ten languages, including "Esperanto," the new world language, which is supposed to have supplanted volapuk.

---

AT a recent meeting of the American Surgical Trade Association in Philadelphia, a resolution was adopted to the effect that all urethral catheters, sounds and bougies would be made in conformity to the French gauge. It will undoubtedly prove desirable to have a uniform scale in force. The change is to take place after this year.

---

THE *Semaine Méd.*, in a recent issue, states that the town of Hagen, in Prussia, has been contemplating changing the names of some of its streets to do honor to living celebrities. Two of the names proposed are those of physicians, von Behring and Koch, and Roentgen's name was also suggested. In advocating the measure, one of the speakers remarked that it would please the medical profession to have its members thus honored, but another speaker expressed some doubt on this point, as the three streets in question all lead to the cemetery.—*Journal A. M. A.*

WHEN writing your paper for the State meeting, remember several things; select a subject with which you have had rich and practical experience—any one can read text-books without going a hundred miles, leaving work and business, and paying three dollars a day to hear you read extracts from one; collect your thoughts, clarify and simplify them, boil them down and then write your paper; go over it again and cut out all non-essentials, and all self-evident data—and you will then have a paper from which every one will learn something—and you will go home thoroughly satisfied and proud of your effort.—*Southern Med. and Surg.*

---

THE Seventh Annual Meeting of the American Roentgen Ray Society will be held August 29, 30, 31, 1906, at the Cataract and International Hotels, Niagara Falls, N. Y. A large and interesting program, containing the names of the best known X-ray workers in this country, as well as a number from abroad, has been prepared. An interesting feature of the meeting will be the exhibit of prints and negatives. The railroads have granted a rate of a fare and a third on the certificate plan. The officers of the Society are: President, Dr. Henry Hulst, Grand Rapids, Mich.; Secretary, Dr. Geo. C. Johnston, Pittsburg, Pa.; Treasurer, Dr. Leavitt E. Custer, Dayton, Ohio; Vice-presidents, Dr. Russel H. Boggs, Pittsburg, Pa.; Dr. Clarence E. Skinner, New Haven, Conn.; Dr. Ennion G. Williams, Richmond, Va.; Dr. Eugene W. Caldwell, New York, N. Y. Full information regarding the meeting and application blanks for membership may be obtained by addressing the secretary, Dr. Geo. C. Johnston, 611 Fulton Building, Pittsburg, Pa.

